# Vivisectors and Their Work

**Published:** 1889-04-13

**Authors:** 


*(To be continued.)*

### Within the Wards.

### VIVISECTORS AND THEIR WORK.

*(Continued from page 4.)*

An Experiment for the Reader.

Any reader who wishes to convince himself of this may put the thumb of his right hand to the right side of his neck and the forefinger to the left side until he feels the steady pulsations of the right and left carotid arteries. If now he press them firmly backwards and inwards against his vertebral column, he will soon find himself becoming either faint or drowsy, or both. The reason of his faintness is that by compressing the arteries he has diminished the quantity of blood in the brain. Within the brain the *carotid* gives off as a branch the *middle cerebral artery*. This vessel is so large, and so almost directly in a line with the main trunk, as to be in most cases practically a continuation of it. It thus, of course, receives the full force of each arterial pulsation; and if there is any general weakness of the walls of the arteries in the brain, this particular vessel, or one or more of its branches, will naturally be the first to give way. As a matter of fact, one of the branches of this artery, called the *lenticulo-striate*, has so often given way, that it has been designated by physicians *par excellence* the "artery of hæmorrhage." This artery, then—the *middle cerebral*, with its *lenticulo-striate* branch—was the centre and point of interest in all the elaborate experiments of Messrs. Victor Horsley and Walter Spencer.

Inside the Skull.

When the skullcap and the hard covering (*dura mater*) immediately beneath it were removed, the experimenters carefully observed the state of the brain and circulation in its natural condition. They noticed the colour of the brain substance, and the faint red tinge which the freely coursing blood gave to it. They remarked, too, the pulsations which could be seen following immediately upon each beat of the heart. In the middle cerebral artery, which lay in its natural place in the *Fissure of Sylvius*, the pulsations were especially distinct and could easily have been counted. That was the natural condition. Pressure was now made upon the carotid artery in the neck, and immediately all pulsation ceased in the middle cerebral artery, and the substance of the brain became paler. It was clear, therefore, that the pressure of the trunk artery had effectually checked the flow of blood into the branches, exactly as had been anticipated. The pressure was then removed, with the result that the pulsation immediately returned, and with it the colour proper to the brain when supplied with its normal quantity of blood. The compression and the cessation of compression were repeated again and again, and always with the same result.

What does it Prove?

But, it may be argued, those monkeys on which these experiments were made were not apoplectic, and therefore the operation could prove nothing at all, except what was practically quite as well known before. The monkeys were not apoplectic, it is true; but it was possible to make them worse than apoplectic, and that condition the operators proceeded to bring about. In apoplexy, a blood-vessel is ruptured at one point, and the blood flows out, more or less slowly, at the leaky spot. The vivisectors cut the large middle cerebral artery, with its lenticulo-striate and other branches, clean across, as if they had cut a water-pipe across, instead of making a little hole in its side as the frost does. Then, with each stroke of the heart, the blood pumped out in full jets, and if it had not been promptly stopped the anæsthetised monkey would have died in a very few minutes. This was a condition much worse than apoplexy. To stop the bleeding, recourse was again had to the pressure of the carotid artery in the neck. Again all pulsation ceased; the blood was no longer pumped out of the larger vessels in jets, but merely oozed for a short time, and finally ceased.

*(To be continued.)*

* This list is practically suggested by Mr. F. Clare Melhado, secretary of the Middlesex Hospital.

